# MicroRNA-30c-5p ameliorates hypoxia-reoxygenation-induced tubular epithelial cell injury via HIF1α stabilization by targeting SOCS3

**DOI:** 10.18632/oncotarget.21582

**Published:** 2017-10-06

**Authors:** Yan-Fang Zou, Wei-Tang Liao, Zong-Jie Fu, Qian Zhao, Yong-Xi Chen, Wen Zhang

**Affiliations:** ^1^ Department of Nephrology, Ruijin Hospital, School of Medicine, Shanghai Jiao Tong University, Shanghai 200025, PR China; ^2^ Cellular Differentiation and Apoptosis Laboratory, Key Laboratory of National Ministry of Education, School of Medicine, Shanghai Jiao Tong University, Shanghai 200025, PR China

**Keywords:** hypoxia-reoxygenation, microRNA, apoptosis, hypoxia-inducible factor-1α

## Abstract

The cellular hypoxia-reoxygenation (H/R) model is an ideal method to study ischemia-reperfusion injury, which is associated with high mortality. The role of microRNA-30c-5p (miR-30c-5p) in the H/R epithelial cell model remains unknown. In the current study, we observed a significant reduction in apoptosis when miR-30c-5p was up-regulated. We also found decreased levels of C-caspase-3 (C-CASP3) and Bcl-2-associated X (BAX) proteins and increased levels of B-cell lymphoma-2 (BCL2). Epidermal growth factor receptor (EGFR) showed similar results. Down-regulating miR-30c-5p increased the levels of apoptosis and C-CASP3 and BAX expression; additionally, cell proliferation was inhibited. Hypoxia-inducible factor 1α (HIF1α) protein expression levels were up-regulated in response to up-regulation of miR-30c-5p expression. The anti-apoptotic and proliferative effects of miR-30c-5p decreased significantly after the HIF1α protein levels were knocked down. Using a luciferase reporter assay, we confirmed that miR-30c-5p targets suppressor of cytokine signaling-3 (SOCS3). HIF1α levels increased when SOCS3 was blocked. Our data show that SOCS3 expression enhances apoptosis in the H/R model. In conclusion, up-regulating miR-30c-5p protects cells from H/R -induced apoptosis and induces cell proliferation; furthermore, HIF1α markedly contributes to this protective effect. MiR-30c-5p stabilizes HIF1α expression by targeting SOCS3 to achieve anti-apoptotic and proliferative effects.

## INTRODUCTION

Ischemia-reperfusion-induced kidney injury (IR-AKI) is a common cause of acute kidney injury that leads to high mortality and morbidity [1–3]. Ischemia-induced injury involves hypoxia-related damage to the cells [4, 5]. Given this background, the cellular hypoxia-reoxygenation (H/R) model was used to study ischemia-reperfusion in the current study. IR-AKI progression can be divided into three phases: initiation, extension and repair [6]. The epithelial cells that are lethally injured undergo necrosis or apoptosis, which occurs during the extension phase of IR-AKI [7–10]. Therefore, reducing apoptosis in the extension phase and accelerating proliferation in the repair phase could protect tubular epithelia from hypoxia-induced injury [11, 12].

Our previous study confirmed the value of miR-30c-5p in the diagnosis of acute kidney injury; miR-30c-5p was up-regulated in the cellular H/R model (NRK52E, rat kidney epithelial cells) [13]. Moreover, it has been reported that miR-30c is involved in the apoptosis process of multiple diseases [14–16]. Therefore, the aim of the current study is to explore whether miR-30c-5p is involved in H/R-induced apoptosis and to study its role in repair after hypoxia injury.

Previous studies have reported that hypoxia-inducible factor 1α (HIF1α) is a key transcription factor that protects cells from hypoxia injury [17–19]. HIF1α was also shown to be induced during reperfusion after ischemia and is crucial for the survival of proximal tubular cells [20]. Considering that miR-30c-5p and HIF1α are potentially involved in apoptosis in the H/R model, another aim of the current study is to investigate the interactions between these mediators and to explore the potential mechanisms involved.

## RESULTS

### Renal tubular epithelial cell apoptosis and elevated levels of microRNA-30c-5p are found in the rat ischemia-reperfusion model

**Figure 1:**
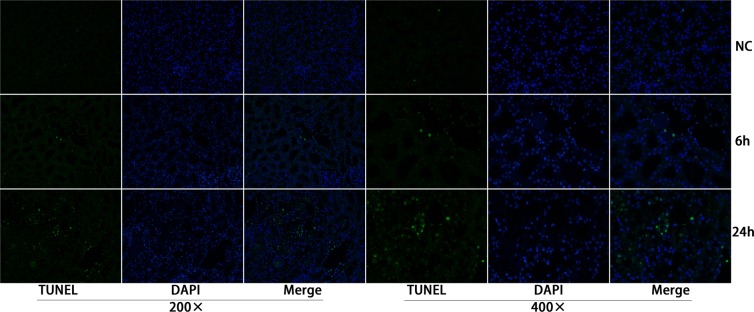
Apoptosis of the renal tubular epithelia cells was induced in the ischemia/reperfusion animal model TUNEL staining showed that apoptosis was detected 6 h post operation and peaked at 24 h after operation.

To investigate whether apoptosis was involved in ischemia-reperfusion kidney injury, TUNEL staining was conducted on fixed kidney tissues from rats that underwent ischemia-reperfusion surgery. Apoptosis was detected at 6 h and peaked at 24 h after the ischemia-reperfusion surgery (Figure 1). At 48-72 h, the apoptosis levels decreased; the results are presented in Supplementary Figure 1.

**Figure 2:**
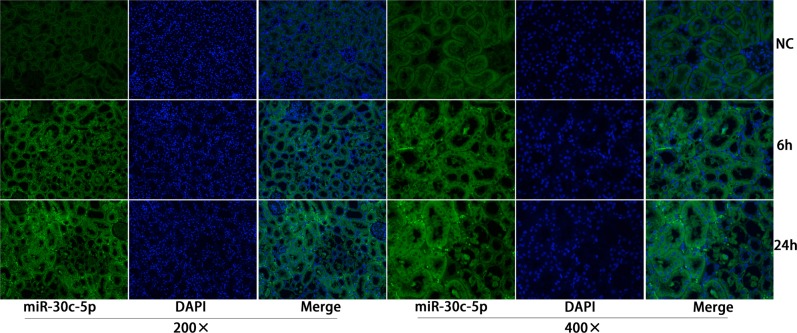
MiR-30c-5p was elevated in the ischemia/reperfusion animal model *In situ* hybridization was performed to examine miR-30c-5p expression in the kidney cortex. MiR-30c-5p was detected in renal tubular and tubule-interstitial cells 6 h post operation and increased at 24 h after operation.

Our previous study showed increased miR-30c-5p levels in the urine samples of rats that underwent I/R surgery. To further study miR-30c-5p expression levels in injured kidney tissues, *in situ* hybridization staining was conducted to detect the expression levels and the location of miR-30c-5p. We detected miR-30c-5p in the renal tubular and tubule-interstitial cells at 6 h after surgery, and its expression peaked at 24 h (Figure 2). Between 48-72 h, miR-30c-5p levels decreased; the results are presented in Supplementary Figure 2. Additionally, we detected the expression levels of miR-30c-5p in the H/R model in HK2 (human kidney, cortex/proximal tubule) cells, and the results showed a significant up-regulation after exposure to 24 h of hypoxia (Supplementary Figure 3).

### MicroRNA-30c-5p alleviates apoptosis and promotes proliferation in cellular hypoxia-reoxygenation injury

**Figure 3:**
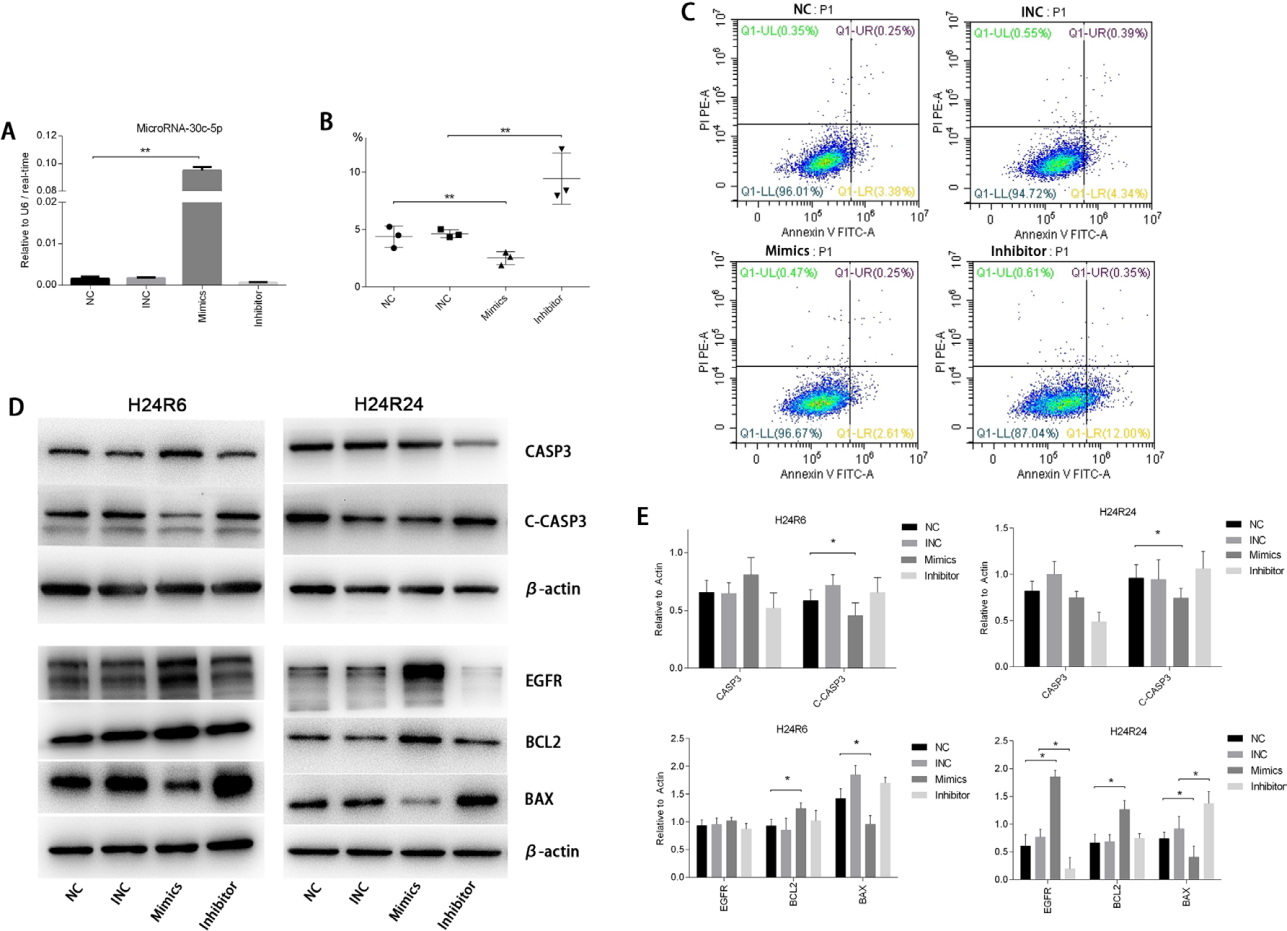
MiR-30c-5p reduced apoptosis and promoted proliferation in cellular H/R injury **(A)** The oligos were transfected into HK2 cells. MiR-30c-5p levels were increased significantly in the cells transfected with the mimics, whereas their levels were not altered in inhibitor-transfected cells. **(B-C)** Flow cytometry was conducted to detect apoptosis in the H/R model. The apoptosis levels decreased significantly in the mimics group compared with the control group and increased significantly in the inhibitor group compared with the control group. **(D)** The protein levels of CASP3, Cleaved-CASP3, EGFR, BCL2, BAX were analyzed in the H/R model while the miR-30c-5p was up-/down-regulated. β-actin was used as the internal control. **(E)** Density levels were analyzed using ImageJ, and the histograms show the average values corrected to β-actin. The data are expressed as the mean±S.E.M, n=3, ^*^P<0.05, ^**^P<0.01 compared with the control group.

To investigate the functions of miR-30c-5p in the cellular injury model, we transfected tubular epithelial cells with miR-30c-5p mimics or inhibitor and later subjected them to hypoxia. Quantitative PCR results showed that the transfection efficiency was high (Figure 3A). Flow cytometry was used to identify apoptosis. The results showed that the transfection of miR-30c-5p mimics deceased apoptosis significantly, and the transfection of the inhibitor significantly increased apoptosis, compared with the control group (Figure 3B and 3C). TUNEL staining showed similar results (Supplementary Figure 4). CASP3 and cleaved-CASP3 protein levels were also measured to determine the effects of the miR-30c-5p mimics on apoptosis. Our results showed that miR-30c-5p mimics decreased cleaved-CASP3 levels significantly in both the H24R6 and the H24R24 groups (Figure 3D and 3E). We also evaluated the changes in the protein levels of the BCL2 family in the cellular injury model. The results showed that BCL2 levels were elevated, and BAX levels were reduced, in the mimics-transfection group. The relative ratio of BCL2 and BAX in the mimics-transfection group differed from that of the NC group in H24R24 (Figure 3D and 3E).

**Figure 4:**
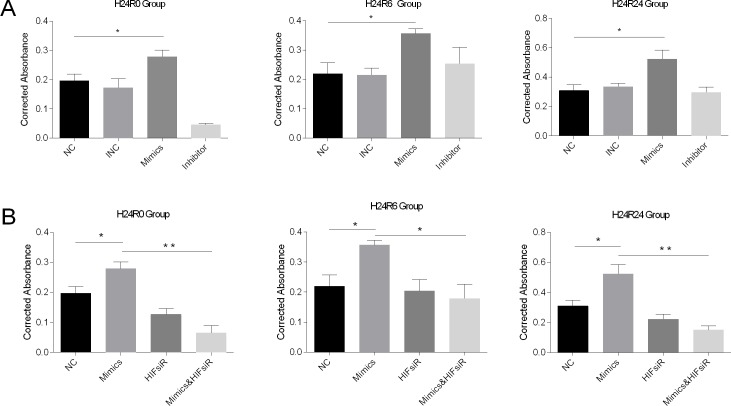
Cell proliferation levels were detected using the CCK-8 assay at 0-, 6- and 24-h time points during cellular H/R injuries (the histograms show corrected absorbances) **(A)** Cell proliferation was promoted in the mimics group and showed no changes in the inhibitor group at 6 h and 24 h. **(B)** Cell proliferation was promoted in the mimics group and was inhibited in the HIFsiR and the Mimics&HIFsiR groups. The data are expressed as the mean±S.E.M, n=3, ^*^P<0.05, ^**^P<0.01 compared with the control group. The Mimics&HIFsiR group was co-transfected with both the mimics and the HIF1α-siRNA.

We evaluated cell proliferation using the CCK-8 assay. The results showed that proliferation was induced in the miR-30c-5p-mimics group (Figure 4A) but that transfection with the inhibitor did not block cell proliferation. Then, we examined EGFR protein levels in the cellular injury model. The Western blot analysis results showed that the EGFR expression levels were elevated in the mimics-transfection group but the levels in cells in the H24R24 group transfected with inhibitor remained unchanged (Figure 3D and 3E).

### MicroRNA-30c-5p stabilizes HIF1α expression in cellular hypoxia-reoxygenation injury

**Figure 5:**
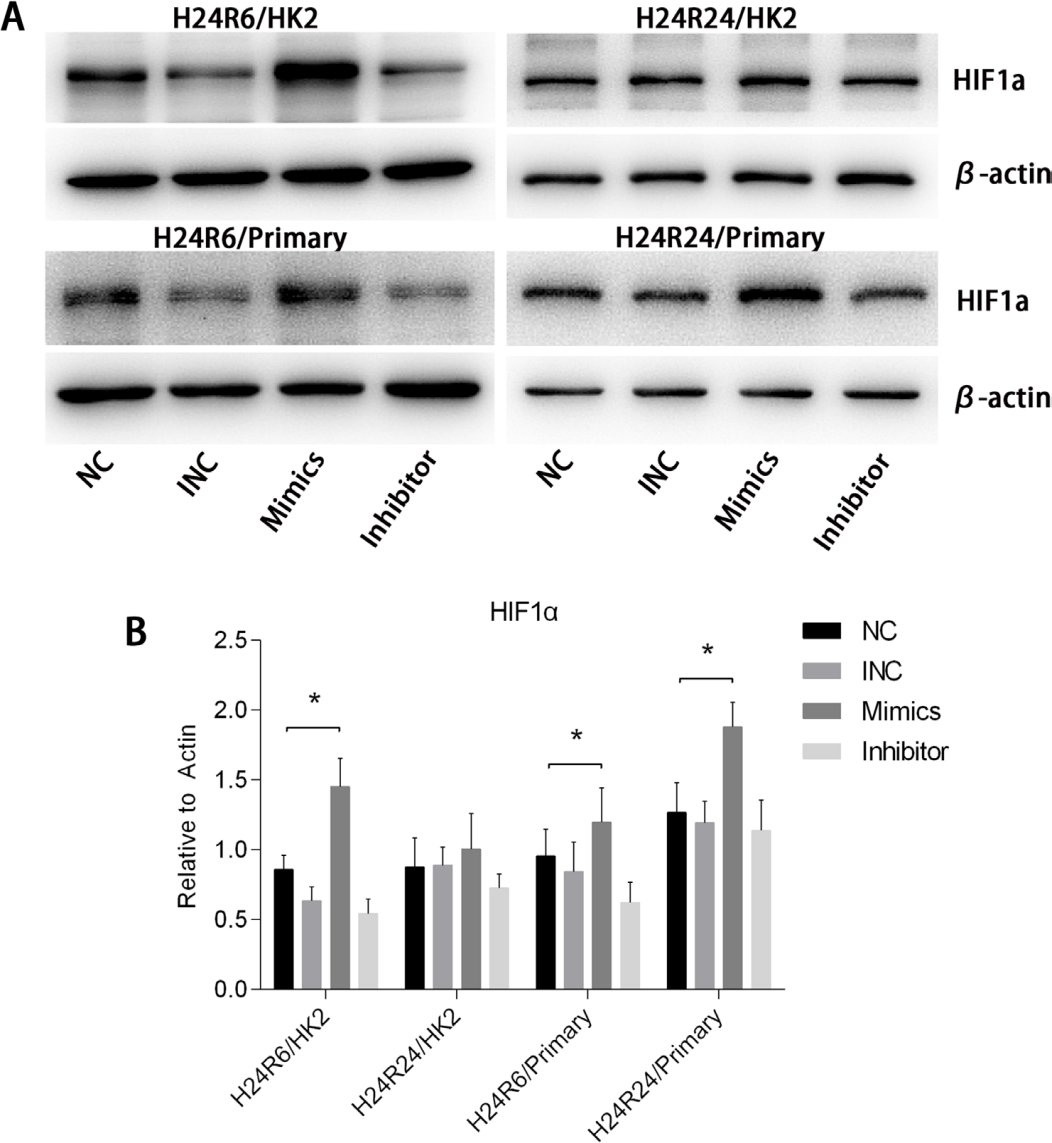
HIF1α expression levels were enhanced by the up-regulation of miR-30c-5p **(A-B)** HIF1α protein levels were examined in the HK2&HRPTEpiC cell model at 6 h and 24 h. HIF1α expression levels were increased in the mimics group, but showed no changes in the inhibitor group. The data are expressed as the mean±S.E.M, n=3, ^*^P<0.05, compared with the control group. Primary human renal proximal tubular epithelial cells (HRPTEpiC).

To explore the effects of miR-30c-5p on HIF1α, we transfected tubular epithelial cells with miR-30c-5p mimics or inhibitor and then exposed them to 1% O_2_. Western blot analysis results indicated that HIF1α levels in the mimics group was higher than those in the control and inhibitor groups at both H24R6 and H24R24 time points (Figure 5). To confirm the effects of miR-30c-5p on HIF1α, we detected HIF1α expression levels in the cellular H/R model using human renal proximal tubular epithelial cells (HRPTEpiC). The results suggested that HIF1α levels in the mimics group were higher than those in the control and the inhibitor groups in the H24R6 and H24R24 groups. We examined the mRNA levels of HIF1α at all time points, including H24R0, H24R6, H24R24, in the mimics-, inhibitor- and control-transfection groups, and no significant changes were found (data not shown).

### HIF1α is required in the repair functions of miRNA-30c-5p mimics in cellular hypoxia-reoxygenation injury

**Figure 6:**
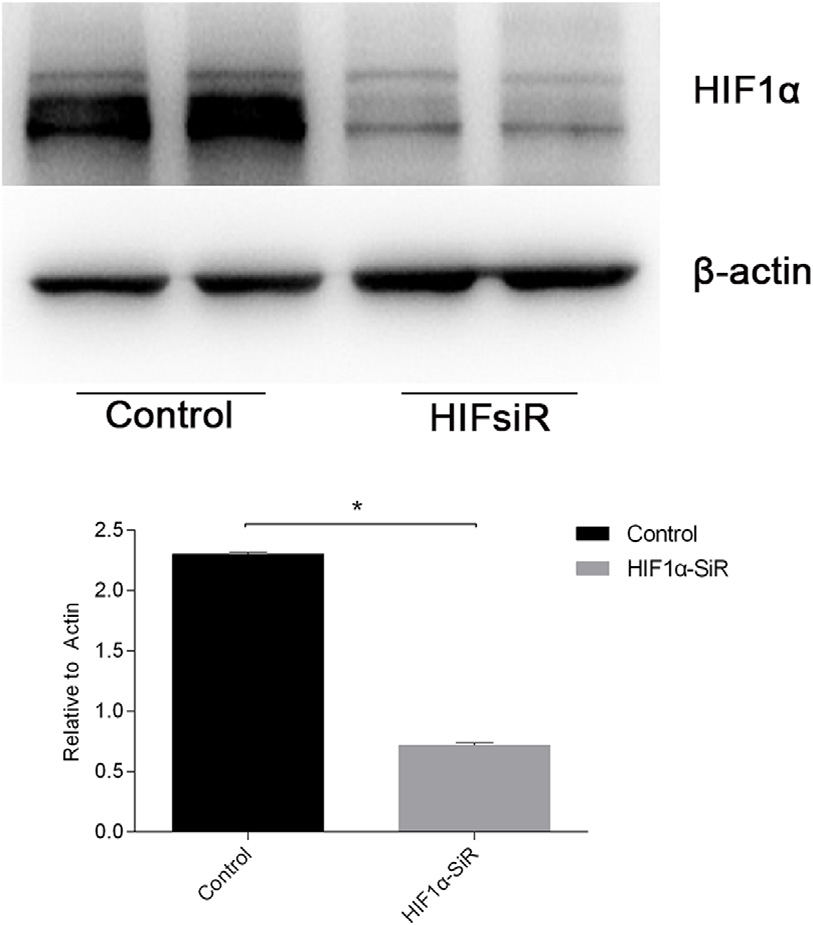
The knockdown efficiency of HIF1α-siRNA was evaluated HIF1α expression levels (protein levels are shown in the top panel and the mRNA levels on the bottom panel) declined sharply in cells that were transfected with HIF1α-siRNA. The data are expressed as the mean±S.E.M, n=3, ^*^P<0.05, compared with the control group.

**Figure 7:**
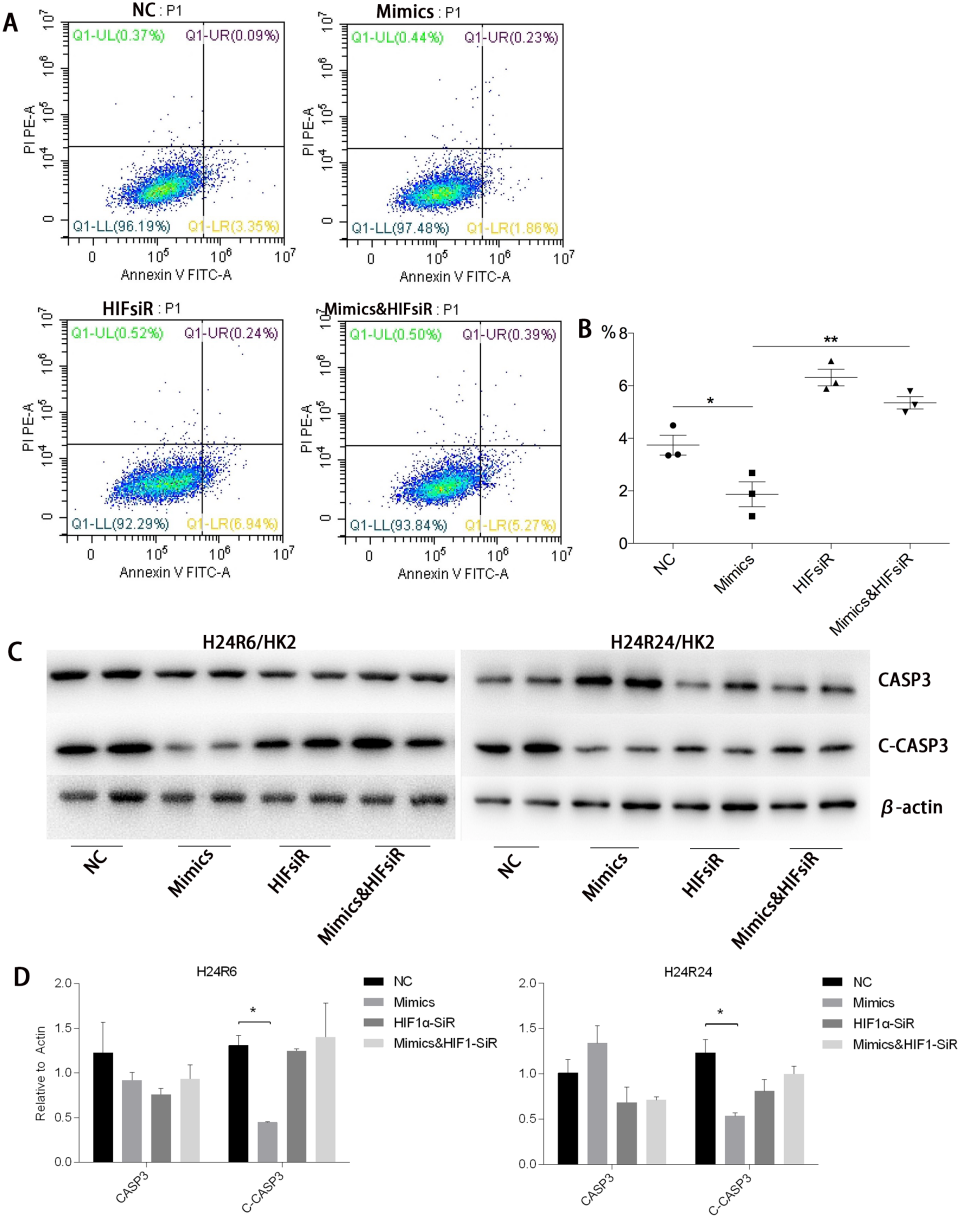
HIF1α was critical for the anti-apoptosis effects of miR-30c-5p **(A-B)** Flow cytometry results confirmed that apoptosis decreased in the mimics group and increased significantly in the HIFsiR and Mimics&HIFsiR groups. **(C-D)** Protein levels of CASP3 and Cleaved-CASP3 were analyzed in the H/R model, while the miR-30c-5p was up-regulated and/or HIF1α was inhibited. β-actin was used as the internal control. The data are expressed as the mean±S.E.M, n=3, ^*^P<0.05, ^**^P<0.01 compared with the control group.

To study whether the protective effects of miR-30c-5p were associated with HIF1α, we inhibited HIF1α expression by transfecting the cells with HIF1α-siRNA. The inhibition efficiency was determined prior to the formal study (Figure 6). Flow cytometry was conducted to detect the differences in apoptosis levels between the cells transfected with miR-30c-5p mimics, HIFsiR and Mimics&HIFsiR. Compared with the HIFsiR and Mimics&HIFsiR groups, the miR-30c-5p-mimics group showed lower levels of early apoptosis. However, the apoptosis levels in the mimics group increased significantly when HIF1α was knocked down (Figure 7A and 7B); TUNEL staining showed similar results (Supplementary Figure 5). Western blot analysis results (Figure 7C and 7D) suggested that cells transfected with miR-30c-5p mimics (mimics group) showed lower expression levels of cleaved-CASP3 than the cells co-transfected with miR-30c-5p mimics and HIF1α-siRNA (Mimics&HIFsiR group).

Additionally, we measured cell proliferation in each group using the CCK-8 assay. The results showed that the pro-proliferation effects of miR-30c-5p mimics disappeared when HIF1α was knocked down (Figure 4B).

### SOCS3 is a target of has-miR-30c-5p and inhibits HIF1α expression

**Figure 8:**
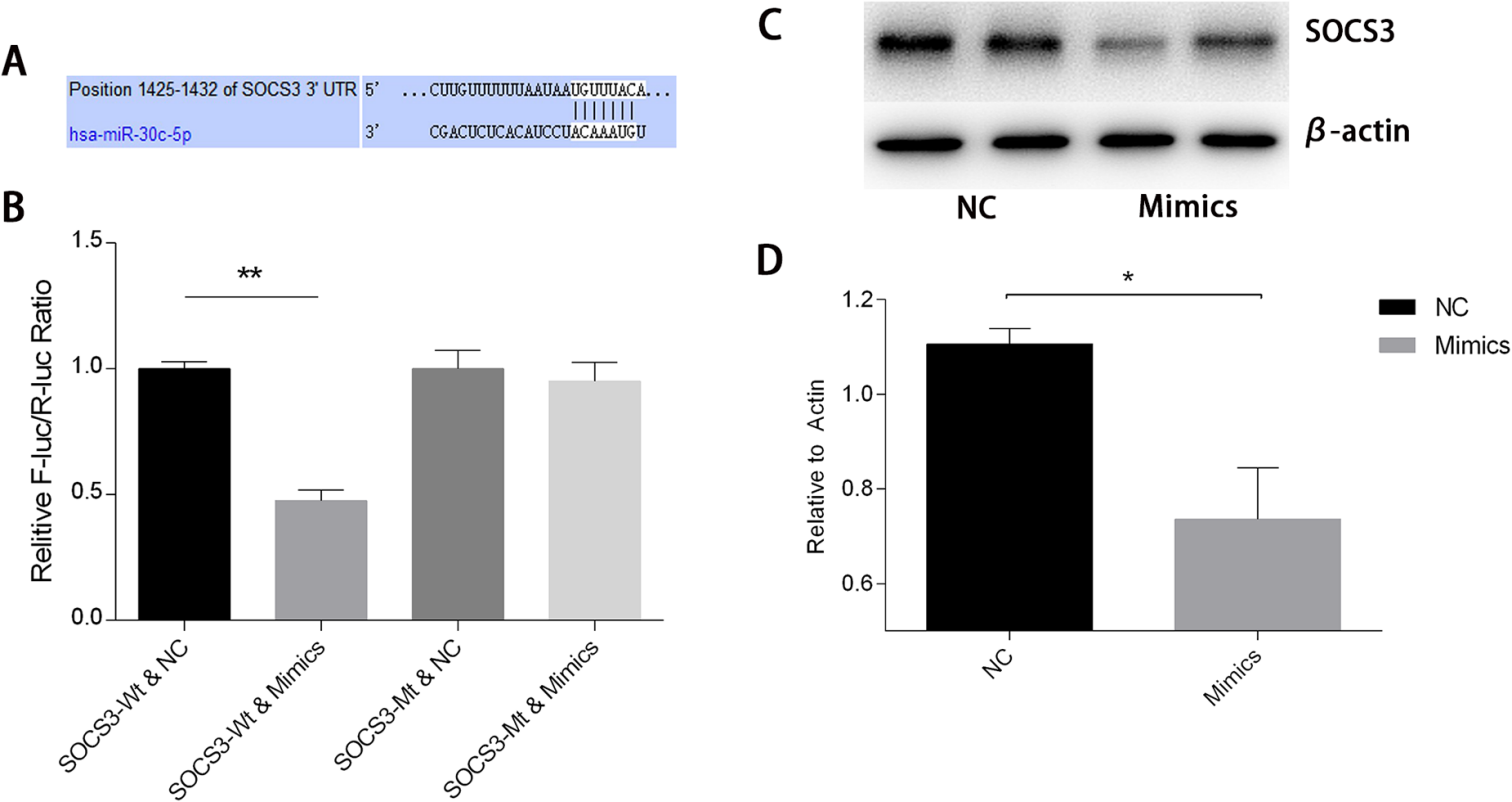
MiR-30c-5p targets the SOCS3 3′UTR to reduce its expression **(A)** Bioinformatics analysis of the predicted interactions between miR-30c-5p and 3′UTR of SOCS3. **(B)** Dual luciferase reporter assay was conducted to demonstrate the interactions between the two. Overexpression of miR-30c-5p diminished fluorescence when the cells were transfected with a wild-type SOCS3-3′UTR vector. **(C-D)** Protein levels of SOCS3 decreased significantly in the miR-30c-5p-overexpression group. The data are expressed as the mean±S.E.M, n=3, ^*^P<0.05, ^**^P<0.01 compared with the control group. SOCS3, suppressor of cytokine signaling-3; SOCS3-WT, wild-type SOCS3-3′UTR vector; SOCS3-MT, mutant-type SOCS3-3′UTR vector.

**Figure 9:**
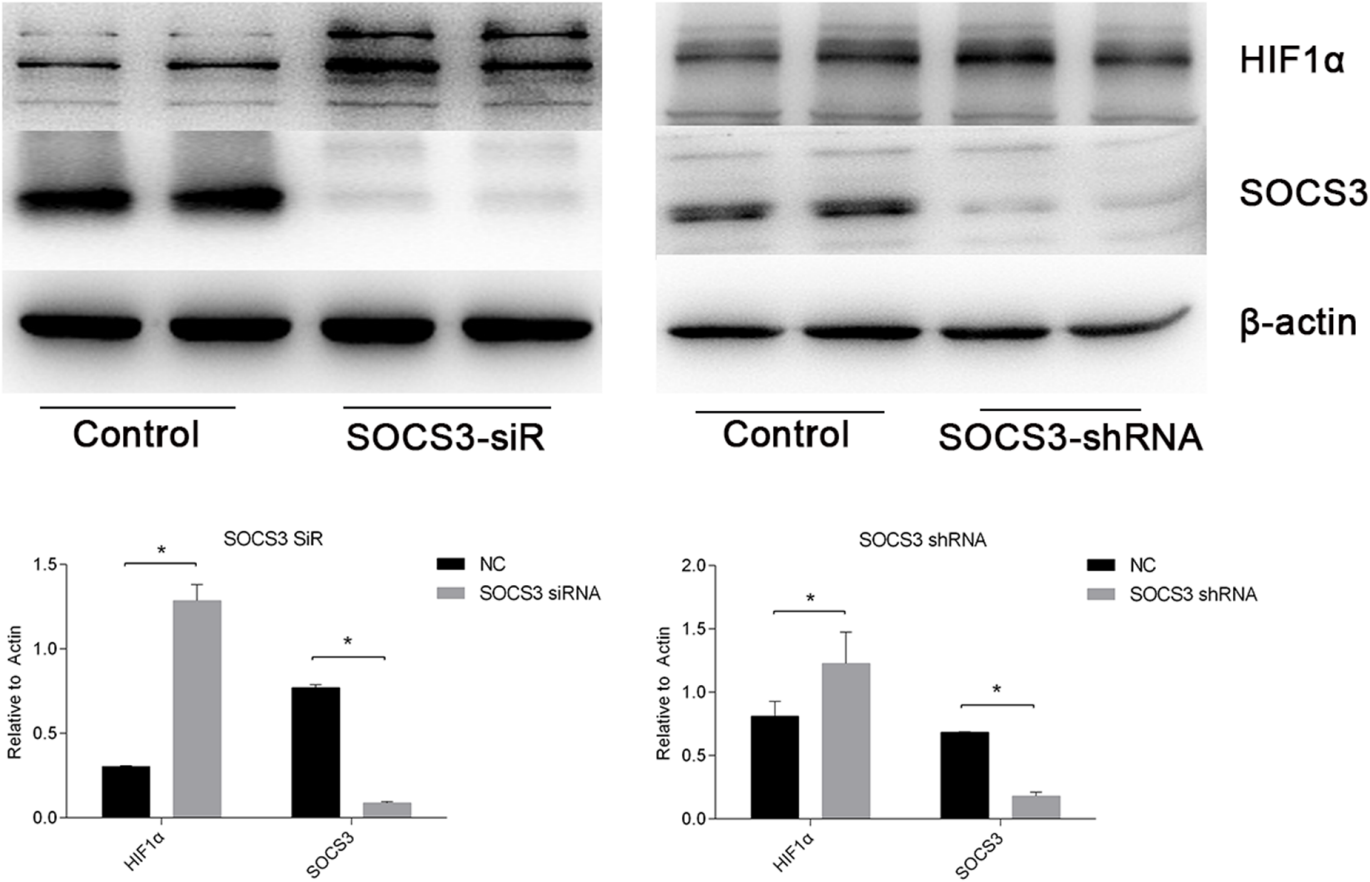
HIF1α expression levels were enhanced when SOCS3 was inhibited siRNA and shRNA were used to inhibit SOCS3. The data are expressed as the mean±S.E.M, n=3, ^*^P<0.05, compared with the control group.

To study the mechanisms involved in the interactions between miR-30c-5p and HIF1α, we predicted the targets of miR-30c-5p using the TargetScan/miRBase database. The prediction found that miR-30c-5p could recognize the 3′UTR of SOCS3 as a target site (Figure 8A). Subsequently, we used the luciferase reporter assay to validate the predicted targets. The results indicated that miR-30c-5p targeted the 3′UTR of SOCS3 and decreased its expression (Figure 8B). We observed a significant decrease in SOCS3 protein levels in the miR-30c-5p-overexpression group (Figure 8C and 8D). Then, we inhibited SOCS3 expression levels and measured HIF1α expression. The inhibition efficiency of the siRNA and shRNA transfections was high. We found that HIF1α levels were increased in the SOCS3-siRNA and the SOCS3-shRNA groups (Figure 9).

### SOCS3 expression promotes apoptosis in cellular hypoxia-reoxygenation injury

**Figure 10:**
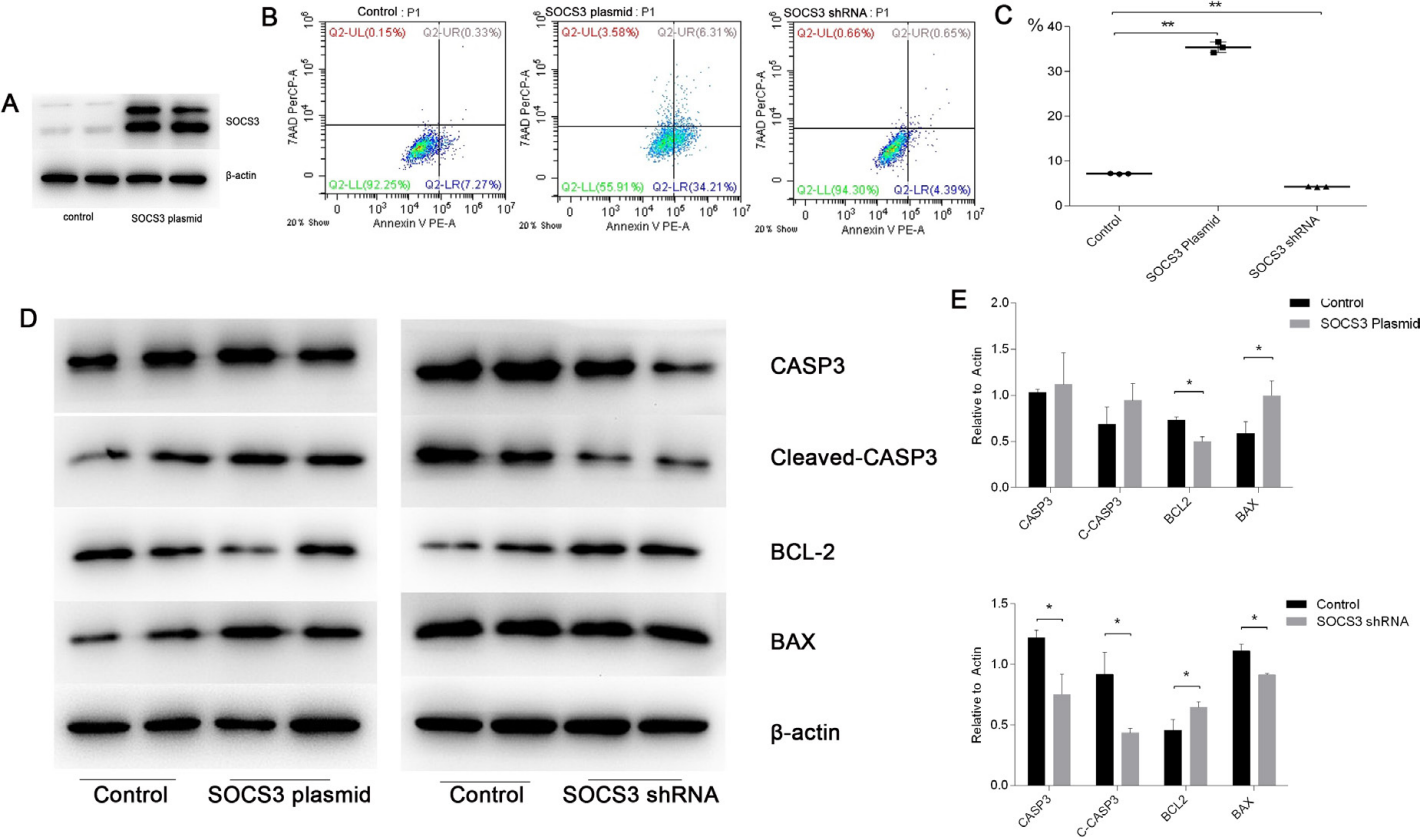
SOCS3 overexpression promoted apoptosis in cellular H/R injuries, whereas SOCS3 inhibition showed the opposite effect **(A)** The transfection efficiency of the SOCS3 overexpression plasmid was high. **(B-C)** Flow cytometry confirmed that apoptosis increased in the SOCS3-overexpression group and decreased significantly in the SOCS3-inhibition group. **(D-E)** Protein levels of CASP3, Cleaved-CASP3, BCL2 and BAX were analyzed in the H/R model with SOCS3 up- or down-regulation. β-actin was used as the internal control. The data are expressed as the mean±S.E.M, n=3, ^*^P<0.05, ^**^P<0.01 compared with the control group.

To illustrate the role of SOCS3 in the cellular model, we analyzed apoptosis levels after SOCS3 up- or down-regulation. The results of preliminary studies indicated that plasmid transfection could up-regulate SOCS3 protein levels significantly (Figure 10A). Flow cytometry results showed that SOCS3 up-regulation promoted apoptosis, whereas SOCS3 down-regulation had the opposite effect (Figure 10B and 10C). We also detected apoptosis-associated protein levels to verify our results. We found that SOCS3 overexpression led to an increase in cleaved-CASP3 and BAX and a decrease in BCL2 levels. SOCS3 inhibition had the opposite effect (Figure 10D and 10E).

### STAT3 is activated in response to cellular hypoxia-reoxygenation injury

**Figure 11:**
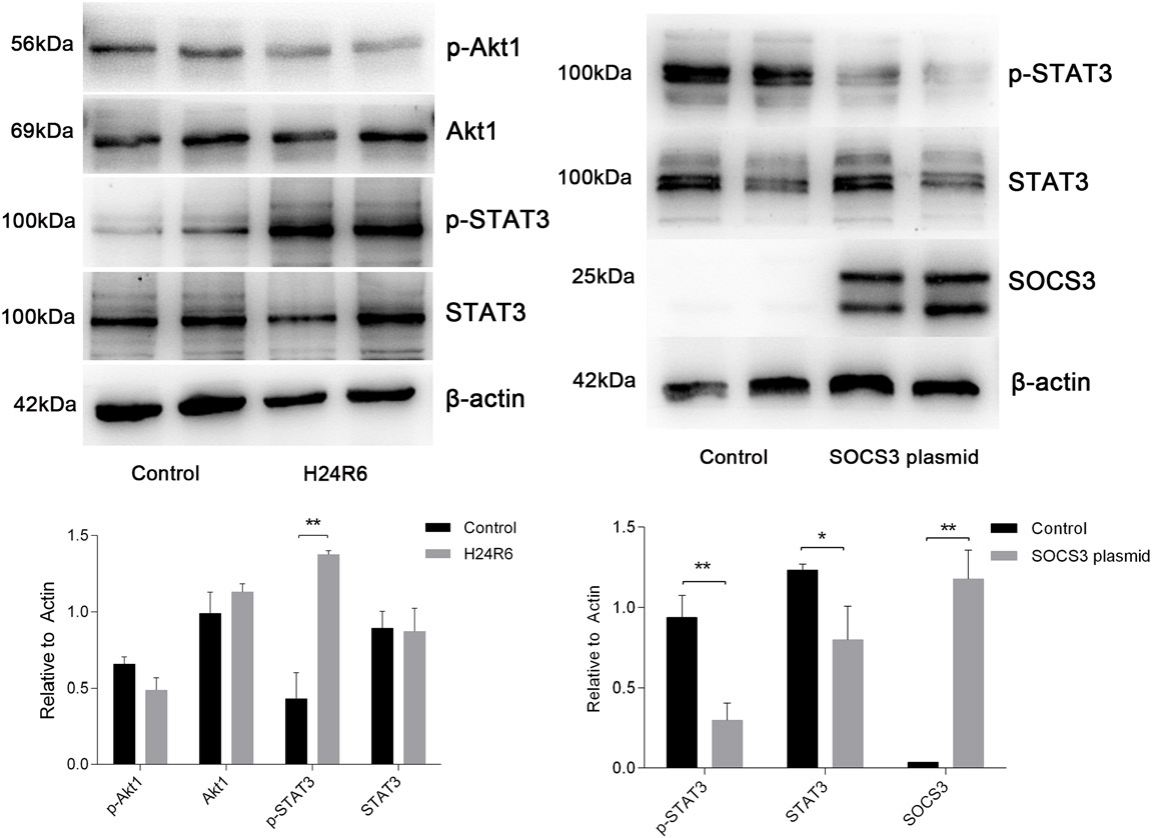
STAT3 was activated in response to cellular H/R injury and was inhibited in cells overexpressing SOCS3 (Left) Protein levels of STAT3, p-STAT3, Akt and p-Akt were analyzed in the H/R model. (Right) Protein levels of STAT3 and p-STAT3 were analyzed in SOCS3 overexpression group. β-Actin was used as the internal control. The data are expressed as the mean±S.E.M, n=3, ^*^P<0.05, ^**^P<0.01 compared with the control group.

To investigate the pathway involved in the response to H/R injury, we examined STAT3 and Akt1 activation. The results showed that the levels of STAT3 phosphorylation were increased in the H24R6 group but that total STAT3 expression was not significantly changed. However, the levels of Akt1 and phosphorylated Akt1 did not exhibit significant changes in the H24R6 group. Next, we examined STAT3 activation in the SOCS3 overexpression group. The results indicated that the levels of STAT3 and phosphorylated STAT3 were decreased in SOCS3 overexpression group. Detailed data are presented in Figure 11.

## DISCUSSION

Our previous studies have demonstrated the diagnostic value of miR-30c-5p for acute kidney injury, and we found that miR-30c-5p levels were increased in the cellular H/R model (NRK52E) [13]. Therefore, we hypothesized that miR-30c-5p may be involved in the pathophysiological process of kidney injury. Given this background, we first examined miR-30c-5p expression in the animal kidney injury model and then measured its expression in the HK2 cellular H/R model. We found that miR-30c-5p levels were increased in both models. We examined the effects of miR-30c-5p on apoptosis and further explored the mechanisms involved. In the current study, we found that miR-30c-5p exerted anti-apoptotic and pro-proliferative activities in the H/R model. Moreover, such effects diminished after the knockdown of HIF1α. Additionally, we demonstrated that miR-30c-5p could target SOCS3, thereby reducing the inhibitory effects of SOCS3 on HIF1α. Using functional assays, we found that higher expression levels of SOCS3 led to an increase in apoptosis.

During the progression of acute kidney injury, renal tubular epithelial cells undergo ischemia and hypoxia, which include a series of events that cause pathological damage, and eventually return to a state of normal tubular epithelium [7, 9]. Apoptosis of tubular epithelial cells is detected during the process [21], which occurs through both caspase-dependent and independent pathways. We confirmed the apoptosis by detecting apoptotic protein levels. In the current study, in addition to Annexin V staining and TUNEL staining, we examined the protein levels of CASP3, cleaved-CASP3, BCL2 and BAX, which confirmed apoptosis.

Several studies on miR-30c-5p have revealed its extensive functions. A study of renal cell carcinoma found that miR-30c-5p could be inhibited by hypoxia and promoted the epithelial-mesenchymal transition (EMT). Another study [22] reported that miR-30c could suppress EMT via blocking the snail1-TGF-β1 pathway and protected against diabetic nephropathy. In addition to EMT, miR-30c-5p was reported to be involved in the apoptotic process [23]. A study on doxorubicin-induced heart failure [24] showed that miR-30 was down-regulated in the myocardium, and this alteration enhanced apoptosis. Based on these findings [23–26], we speculated that miR-30c-5p might be involved in process of apoptosis in hypoxia-induced kidney injury. This speculation was confirmed by our results. In the current study, we found that miR-30c-5p was up-regulated in the model and promoted resistance to apoptosis in epithelial cells. Therefore, we explored the functions of HIF1α in this model and further studied the interactions between HIF1α and miR-30c-5p.

In addition, other targets of miR-30c-5p might be involved in tubular cell injury. NOTCH1 is an important factor in Notch signaling pathway has been reported to be involved in apoptosis in a several disease processes, including kidney injury. NOTCH1 was also reported to be a direct target of miR-30c-5p [27], which might play a role in regulating tubular cell apoptosis. Bnip3L was also reported to be a direct target of miR-30c and is involved in apoptosis of renal tubular epithelial cell in a cisplatin injury model [28]. Moreover, miR-30c could induce apoptosis by targeting the Wnt/β-catenin/BCL9 pathway [16], which promotes apoptosis of tubular cells. Because there might be other targets involved in the effects of miR-30c-5p on apoptosis in the tubular injury model, further study is required to confirm the influence of these targets.

A database analysis showed that miR-30c-5p and EGFR cannot directly bind to one another; therefore, we considered that there might exist an indirect relationship between the two molecules. A study by Peck BC *et al*. [29] showed that inhibiting the miR-30 family resulted to reduced cell proliferation, and Sox9 was confirmed to be a target of the miR-30 family. In addition, Capaccione KM *et al*. [30] reported that Sox9 acts as a hub to control NF-κB and EGFR signaling. Grimont A *et al*. [31] also observed that Sox9 (as a transcription factor) could control EGFR signaling in pancreatic cancer. Thus, we considered that miR-30c might regulate EGFR by acting on Sox9, and future studies would evaluate the relationship between these two factors.

HIF1α is a key transcription factor in ischemia- or hypoxia-induced disease [32]. Previous studies on AKI have shown that HIF1α is involved in disease progression and could protect epithelial cells from hypoxia or toxic stimulus-induced injury [20, 33]. Our study also found that HIF1α could trigger tubule repair during the process of kidney injury [34]. In the current study, HIF1α protected tubular cells from hypoxia-induced apoptosis and could be a key factor in the protective effects of miR-30c-5p on tubular cells. Thus, our results demonstrated the benefits of HIF1α during kidney injury. The results of our study were consistent with the findings in previous reports. Additionally, we found that up-regulation of miR-30c-5p could stabilize HIF1α expression. Due to the negative regulation of miRNA on its target, no direct interactions were found between miR-30c-5p and HIF1α. Therefore, we analyzed miR-30c-5p targets to find the intermediary factors between these two factors.

Predictions from the database indicated that there might be a locus between miR-30c-5p and the 3′UTR of SOCS3. Moreover, prior studies [23, 35] have found that these molecules are associated. We confirmed the relationship between these targets in this study. Moreover, our study found that SOCS3 could block HIF1α expression. However, the mechanisms of their interaction are not completely understood. In general, SOCS3 is considered to be induced by activated STAT3 and in turn, it inhibits the STAT3 pathway [36]. A study [37] on liver regeneration reported that decreased SOCS3 levels enhance IL/STAT3 signaling, thereby promoting liver regeneration and repair. However, a report from Wan *et al.* [38] also found that SOCS3 blocked HIF1α through Akt activation instead of STAT3 in human small lung cancer. In the current study, STAT3 but not Akt1 was activated in hypoxic cells, and this activation could be inhibited by SOCS3 overexpression. Therefore, we speculate that decreased SOCS3 promotes HIF1α activity via activation of STAT3.

In conclusion, we found that miR-30c-5p was up-regulated in the H/R model and could protect epithelial cells from hypoxia-induced injury, while HIF1α markedly contributed to this process. Moreover, miR-30c-5p stabilized HIF1α expression levels by targeting SOCS3 to achieve its anti-apoptosis effects and to promote proliferation. Therefore, miR-30c-5p could be considered a potential novel therapeutic target for kidney injury.

## MATERIALS AND METHODS

### Ischemia-reperfusion-induced rat kidney injury model

As described previously [13, 39], male Sprague-Dawley rats weighing 180 to 200 g were purchased from the Animal Center of Shanghai Institutes for Biological Sciences, Chinese Academy of Sciences, Shanghai, China. Bilateral I/R surgery was performed on the rats in the I/R group for up to 45-minutes, and sham surgery was performed on the animals in the control group. The kidney cortex tissues were fixed in 10% neutral buffered formalin solution for immunofluorescence staining.

### Cell culture and the hypoxia-reoxygenation (H/R) model

HK-2 (human kidney tubular epithelial cells, cortex/proximal tubule) cells were purchased from ATCC (CRL-2190, USA) and cultured in Dulbecco's modified Eagle medium (Gibco) containing 10% fetal bovine serum (FBS, Gibco). HRPTEpiC (human renal proximal tubular epithelial cells) cells were purchased from ScienCell (4100) and cultured in Epithelial Cell Medium (4101, ScienCell) containing 2% FBS (ScienCell) and 1% E piCGS. The cells were routinely cultured according to the suppliers’ recommendations. To establish the H/R model, the cells were grown to monolayers that were 50-60% confluent and subjected to a 24-h period of starvation (in the absence of FBS). Subsequently, the cells were cultured in a hypoxic incubator (1% O_2_, 5% CO_2_, and 94% N_2_, Thermo Electron) as a monolayer at 60% to 80% confluency for 24 h. For the reoxygenation step, the culture medium was replaced with fresh medium containing FBS, and the culture flasks were moved to normoxic conditions. Samples were collected at the following time points: 0 h after hypoxia for 24 h (H24R0); 2 h after hypoxia for 24 h (H24R2); 6 h after hypoxia for 24 h (H24R6); 12 h after hypoxia for 24 h (H24R12); and 24 h after hypoxia for 24 h (H24R24).

### Oligo synthesis and transfection

MicroRNA-30c-5p mimics and inhibitors and negative control oligos were synthesized by GenePharma (Shanghai, China). Small interfering RNAs targeting HIF1α (HIF1α-siRNA-1, HIF1α-siRNA-2, and HIF1α-siRNA-3) and a corresponding negative control were designed and purchased from RiboBio. The cells were cultured in 6-well plates; complete growth medium was removed when the cells reached 50-60% confluence, and Opti-MEM (Gibco) medium was added. The oligos were diluted in Opti-MEM and incubated with the diluted Lipofectamine RNAiMAX Reagent (Invitrogen) for 10 min according to the manufacturer's protocol. The oligo-lipid complexes were added to the wells, and siRNAs were added to the wells at a concentration of 20 pmol per well. The medium was replaced by fresh medium after 24 h, and the plates were moved to the hypoxic chamber for the H/R procedure.

### Plasmid construction and transfection

The suppressor of cytokine signaling-3 (SOCS3)-overexpression plasmid (H6252) and the control plasmid (H149) were designed and constructed by Obio Technology (Shanghai, China). Lipofectamine 3000 Transfection Reagent (Invitrogen) was used for the transfection of the SOCS3 plasmid according to the manufacturer's instructions. For SOCS3 silencing, we designed three pairs of shRNAs for SOCS3, incorporated them into lentiviral vectors (Addgene, USA) and generated viral particles using viral packaging technology. The knockout efficiencies were determined for further experiments. The shRNA-SOCS3 sequence with the strongest inhibitory effect was as follows: forward, 5′- CCGGGCTAAGAGATTCGCCTTAAATCTCGAGATTTAAGGCGAATCTCTTAGCTTTTTG-3′; and reverse, 5′- AATTCAAAAAGCTAAGAGATTCGCCTTAAATCTCGAGATTTAAGGCGAATCTCTTAGC-3′.

### Flow cytometry

The cells were cultured in 6-well plates, digested using trypsin, and washed with PBS. Subsequently, the cells were stained with Annexin V-FITC (BD Pharmingen™) and incubated at 4°C for 10 min. The cells were then stained with propidium iodide and washed using PBS. In addition, FSC and SSC were used to distinguish the gated cells by flow cytometry (FACSCalibur, USA).

### Immunofluorescence staining and *in situ* hybridization

Paraffin-embedded kidney sections (4-μm thick) were dewaxed using dimethylbenzene and a graded series of ethanol, and the antigens were retrieved using proteinase K. The samples were permeabilized for subsequent staining. A TdT-mediated dUTP Nick-End Labeling (TUNEL) kit (Roche) was used to stain the tissues, and the samples were incubated at 37°C for 2 h. The nuclei were then stained with DAPI and viewed under an inverted fluorescence microscope (NIKON DS-U3).

Paraffin-embedded kidney sections, 4-μm thick, were dewaxed using dimethylbenzene and graded ethanol and digested with citrate buffer. The samples were then hybridized with 5 ng/μl of the hybridization probe (Rno-miR-30c-5p: 5′-GCTGAGAGTGTAGGATGTTTACA-3′; designed and synthesized by Servicebio, Wuhan, China) overnight. The nuclei were stained with DAPI and viewed with an inverted fluorescence microscope (NIKON DS-U3).

### Western blot analysis

The cells were washed with PBS and lysed in lysis buffer containing 4% sodium dodecyl sulfate (SDS), 20% glycerol, 100 mM dithiothreitol (DTT), and Tris-HCl, pH 6.8, as reported previously [34]. A total of 30-40 μg of the supernatant proteins was loaded and separated by 8-15% sodium dodecyl sulfate-polyacrylamide gel electrophoresis. The samples were incubated with primary antibodies overnight at 4°C after the proteins were transferred to a polyvinylidene fluoride (PVDF) membrane (Millipore, 0.2 μm or 0.45 μm, as appropriate), following the electrophoresis. The primary antibodies were used at dilutions of 1:5000 for β-actin (Sigma), 1:1000 for HIF-1α (Abclonal), 1:2000 for BCL2 (Abclonal), 1:2000 for BAX (Abclonal), 1:2000 for EGFR (Abclonal), 1:2000 for CASP3 (Cell Signaling Technology), 1:2000 for cleaved-CASP3 (Cell Signaling Technology) 1:2000 for SOCS3 (Abclonal), 1:2000 for STAT3 (signal transducer and activator of transcription 3) (Abclonal), 1:2000 for p-STAT3 (Abclonal), 1:2000 for Akt1 (RAC-alpha serine/threonine-protein kinase) (Abclonal), and 1:2000 for p-Akt1 (Abclonal). Then, the membranes were incubated with HRP-conjugated secondary antibodies (Cell Signaling Technology) at room temperature for 2 h. The proteins were visualized with an ECL Chemiluminescent kit (Novex^TM^) and quantified by grayscale analysis using ImageJ software (National Institutes of Health). β-Actin was used as an internal control for all the Western blots.

### RNA extraction, reverse transcription, and quantitative real-time PCR

Total RNA was extracted from cells using Trizol (Ambion) according to the manufacturer's instructions. For the miRNA analysis, an miScript II RT Kit (Qiagen) was used to conduct reverse transcription with 1 μg of total RNA from each sample. Then, the primer assay for hsa-miR-30c-5p (Hs_miR-30c_2 miScript Primer Assay, Qiagen, 218300) was conducted for the subsequent PCR analysis. U6 was set as the internal control, and the following pair of PCR primers was used: sense, GCTTCGGCAGCACATATACTAAAAT; antisense, CGCTTCACGAATTTGCGTGTCAT. The cDNA Reverse Transcription Kit (Applied Biosystems®) was used for mRNA PCR analysis using 2 μg of total RNA from each sample. β-Actin was used as the internal control. The following PCR primers were used: β-actin sense, CATGTACGTTGCTATCCAGGC and antisense, CTCCTTAATGTCACGCACGAT; and HIF1α sense, GAACGTCGAAAAGAAAAGTCTCG and antisense, CCTTATCAAGATGCGAACTCACA. Real-time PCR was performed using the SYBR® Green PCR Master Mix (Applied Biosystems) and the StepOne Plus Real-Time PCR System (Applied Biosystems, CA, USA) as per the manufacturer's protocols. The relative expression level changes were calculated using the 2^−ΔCt^method.

### Proliferation assay

The cells were cultured in 96-well plates, and fresh medium was added after the oligo transfection and H/R. A total of 10 μl of CCK-8 (Cell counting kit-8, Dojindo, Japan) solution was added into the wells and incubated at 37°C for 1-4 h according to the manufacturer's instructions. The absorbances at 450 nm were measured using a microplate reader (Thermo). The blank absorbance values were subtracted from the readings and subsequent analyses of the readings were conducted.

### Luciferase reporter assay

The targets of miR-30c-5p and their potential binding regions were predicted using the TargetScan database (http://www.targetscan.org/vert_71/). pMIR-REPORT-SOCS3-3′UTR-Wt (H5471), pMIR-REPORT-SOCS3-3′UTR-Mt (H5472); has-miR-30c-5p mimics and scrambled RNA were designed and purchased from Obio Technology (Shanghai, China). HEK293T cells were cultured in 96-well plates until 70% confluent and incubated at 37°C for 24 h. Then, 0.2 μg of SOCS3-3′UTR-Wt or SOCS3-3′UTR-Mt was transfected along with 100 nM has-miR-30c-5p mimics or scrambled RNA, and the medium was replaced with fresh medium after 6 h. Each group had 6 replicates (N=6). After incubating the plates at 37°C for 48 h, the firefly and Renilla luciferase activities were measured using the Dual Luciferase Assay Kit (Promega) according to the manufacturer's instructions.

### Statistical analysis

The data represent the mean±SEM value of the variables. The *t* test was used when comparing the data between two groups, and a one-way ANOVA was used to compare data from three or more groups. When the α level ≤0.05, the differences were considered significant. The SPSS Statistical Software (Version 13.0, SPSS, Inc.) was used to conduct the statistical analyses. The graphs were generated using the GraphPad Prism Software (GraphPad Software Version 6.0c, CA, USA). The grayscale levels of the Western blot images were analyzed using ImageJ software (National Institutes of Health, USA).

## SUPPLEMENTARY MATERIALS FIGURES



## Abbreviations

AKI: acute kidney injury
Akt1: AKT serine/threonine kinase 1
BAX: BCL-2 associated X
BCL-2: B-cell lymphoma-2
BCL-9: B-cell lymphoma-9
CASP3: Caspase-3
CCK8: cell counting kit-8
EGFR: epidermal growth factor receptor
EMT: epithelial-mesenchymal transition
H/R: hypoxia-reoxygenation
H24R0: hypoxia for 24hrs and reoxygenation for 0hrs
H24R6: hypoxia for 24hrs and reoxygenation for 6hrs
HIF1α: hypoxia inducible factor-1α
HIFsiR: HIF1α-siRNA
HRPTEpiC: primary human renal proximal tubular epithelial cells
IR-AKI: ischemia/reperfusion-acute kidney injury
miRNA: microRNA
NC: negative control
NF-κB: nuclear factor kappa-light-chain-enhancer of activated B cells
shRNA: short hairpin RNA
SOCS3: suppressor of cytokine signaling
SOX9: SRY-box 9
STAT3: signal transducer and activator of transcription 3
TUNEL: TdT-mediated dUTP Nick-End Labeling
UTR: untranslated region
