# Supplementary material for: MicroRNA-30c-5p ameliorates hypoxia-reoxygenation-induced tubular epithelial cell injury via HIF1α stabilization by targeting SOCS3
Source: Oncotarget. 2017 Oct 6;8(54):92801–14. doi: 10.18632/oncotarget.21582 (PMC5696223; doi:10.18632/oncotarget.21582)
Supplement: Supplementary file 1 [file oncotarget-08-92801-s001.pdf]

MicroRNA-30c-5p ameliorates hypoxia-reoxygenation-induced tubular epithelial cell injury via HIF1α stabilization by targeting SOCS3

SUPPLEMENTARY MATERIALS

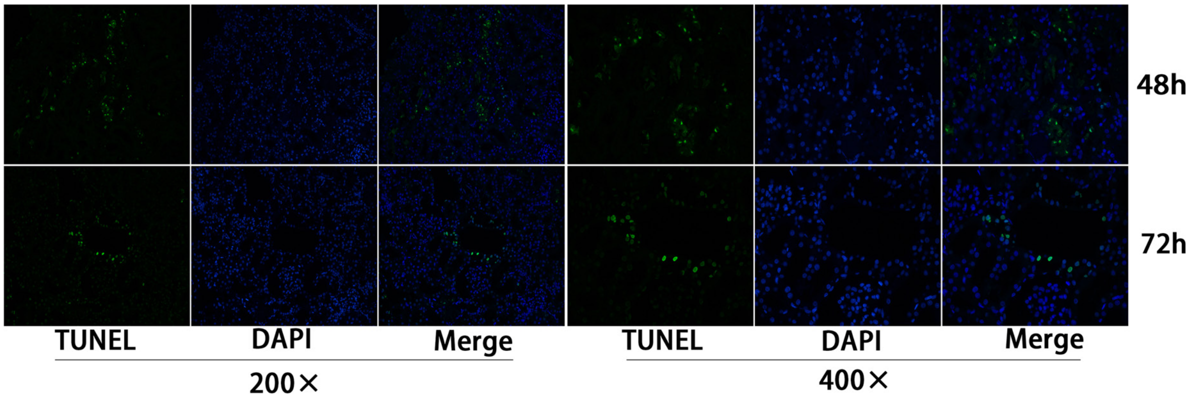

Supplementary Figure 1: Apoptosis of renal tubular epithelia cells was induced in ischemia/reperfusion animal model. TUNEL staining results showed that apoptosis was detected at 48-72 h post operation.

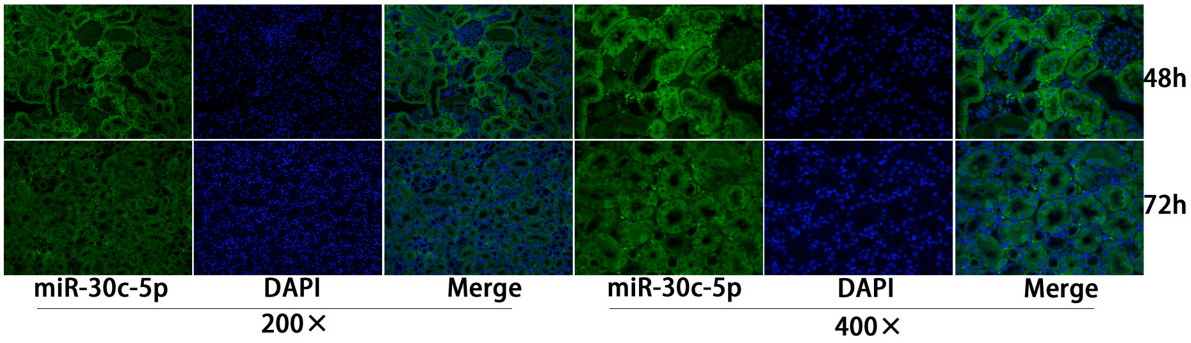

Supplementary Figure 2: MiR-30c-5p was elevated in the ischemia/reperfusion animal model. *In situ* hybridization was performed to examine miR-30c-5p expression levels in the kidney cortexes. MiR-30c-5p levels were detected in the renal tubular and the tubule-interstitial cells at 48-72 h post operation.

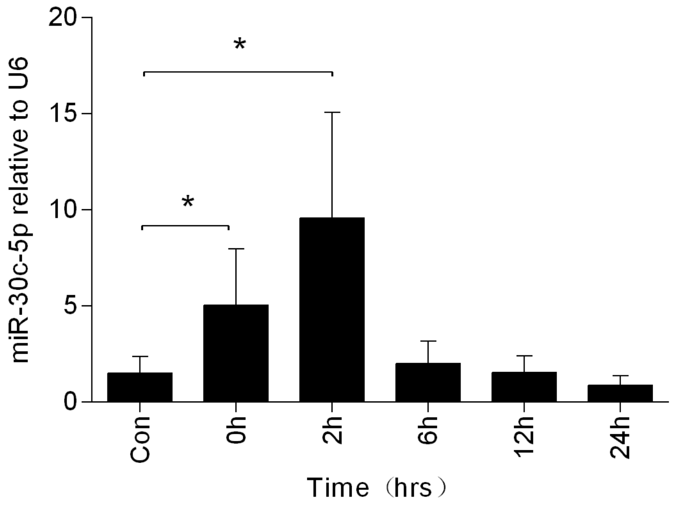

Supplementary Figure 3: MiR-30c-5p was elevated in cellular H/R injuries. Quantitative PCR was performed to examine the miR-30c-5p expression levels in the HK2 cell model. The miR-30c-5p expression levels were elevated at 0-2 h after reoxygenation. The data are expressed as the mean±S.E.M, n=3, $^{*}P<0.05$, compared with the control group.

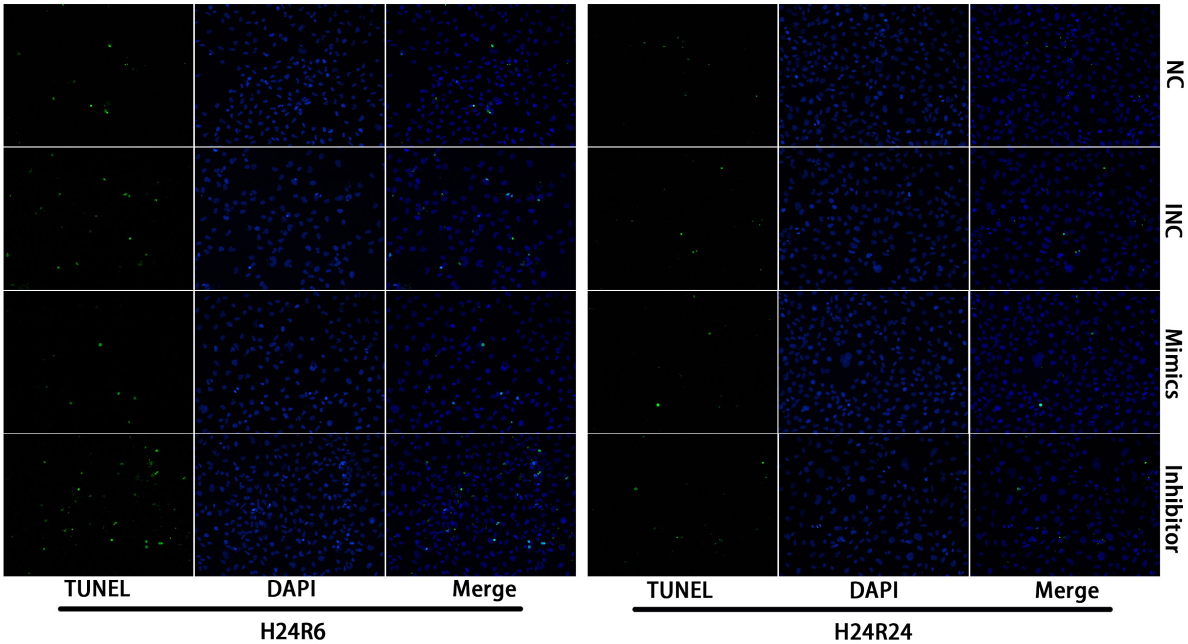

Supplementary Figure 4: MiR-30c-5p was shown to reduce apoptosis in cellular H/R injuries. TUNEL staining showed that apoptosis decreased in the mimics group at the 6-h and 24-h time points.

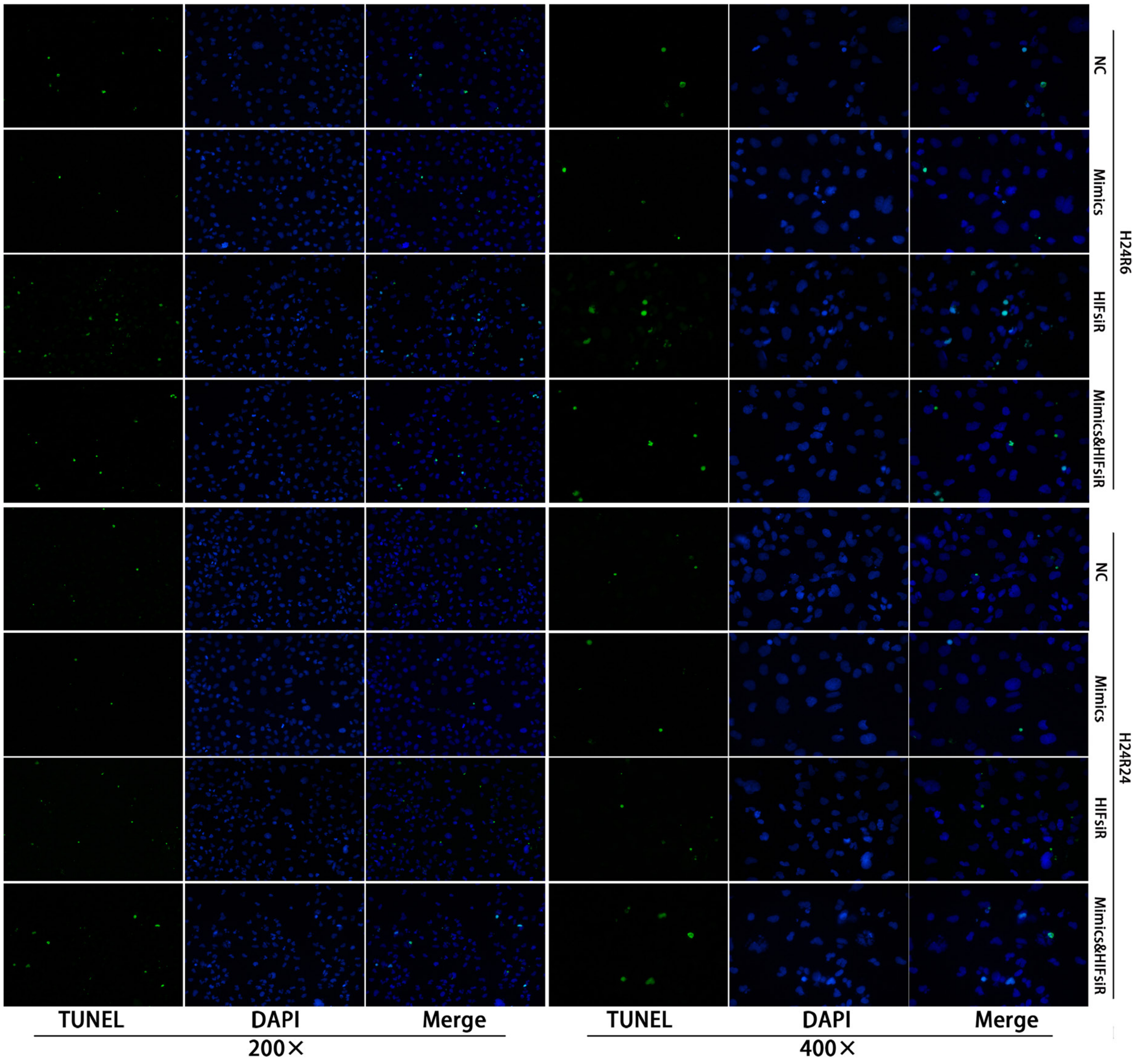

Supplementary Figure 5: HIF1α was critical for the anti-apoptosis effects of miR-30c-5p. TUNEL staining indicated that apoptosis decreased in the mimics group and increased significantly in the HIFsiR group and the Mimics&HIFsiR group.
